# New 4-Aminoproline-Based Small Molecule Cyclopeptidomimetics as Potential Modulators of α_4_β_1_ Integrin

**DOI:** 10.3390/molecules26196066

**Published:** 2021-10-07

**Authors:** Andrea Sartori, Kelly Bugatti, Elisabetta Portioli, Monica Baiula, Irene Casamassima, Agostino Bruno, Francesca Bianchini, Claudio Curti, Franca Zanardi, Lucia Battistini

**Affiliations:** 1 Department of Food and Drug, University of Parma, Parco Area delle Scienze 27/A, 43124 Parma, Italy; andrea.sartori@unipr.it (A.S.); kelly.bugatti@unipr.it (K.B.); elisabetta.portioli@studenti.unipr.it (E.P.); agostino.bruno@unipr.it (A.B.); claudio.curti@unipr.it (C.C.); franca.zanardi@unipr.it (F.Z.); 2 Department of Pharmacy and Biotechnology, University of Bologna, Via Irnerio 48, 40126 Bologna, Italy; monica.baiula@unibo.it (M.B.); irene.casamassima@unibo.it (I.C.); 3 Department of Experimental and Clinical Biomedical Sciences, University of Florence, Viale G.B. Morgagni 50, 50134 Firenze, Italy; francesca.bianchini@unifi.it

**Keywords:** aminoproline scaffold, integrin targeting, ligand design, peptidomimetic synthesis, leukocyte integrins

## Abstract

Integrin α_4_β_1_ belongs to the leukocyte integrin family and represents a therapeutic target of relevant interest given its primary role in mediating inflammation, autoimmune pathologies and cancer-related diseases. The focus of the present work is the design, synthesis and characterization of new peptidomimetic compounds that are potentially able to recognize α_4_β_1_ integrin and interfere with its function. To this aim, a collection of seven new cyclic peptidomimetics possessing both a 4-aminoproline (Amp) core scaffold grafted onto key α_4_β_1_-recognizing sequences and the (2-methylphenyl)ureido-phenylacetyl (MPUPA) appendage, was designed, with the support of molecular modeling studies. The new compounds were synthesized through SPPS procedures followed by in-solution cyclization maneuvers. The biological evaluation of the new cyclic ligands in cell adhesion assays on Jurkat cells revealed promising submicromolar agonist activity in one compound, namely, the *c*[Amp(MPUPA)Val-Asp-Leu] cyclopeptide. Further investigations will be necessary to complete the characterization of this class of compounds.

## 1. Introduction

Integrins constitute a major class of cell adhesion receptors in mammals and play a vital role in cell–cell and cell–extracellular environment communication by regulating crucial aspects of cellular functions, including migration, adhesion, differentiation, growth, and survival. They are expressed in almost all cell types with varied distribution pattern [1,2]. Given their fundamental contribution in human physiology, specific integrin dysregulation phenomena are linked to the pathogenesis of many disease states (including cancer, thrombosis, vascular diseases, autoimmune pathologies, osteoarthritis, osteoporosis), and this renders them attractive targets for biomedical research [3,4,5].

The integrin family comprises 24 different heterodimeric subtypes, classified according to the specific, non-covalent combination between α and β subunits. Among these, the α_4_β_1_ and α_4_β_7_ subtypes, as well as the β_2_ integrin subclass, belong to the leukocyte-specific integrin family and are involved in the modulation of immune functions. In particular, the α_4_β_1_ integrin, also known as very late antigen-4 (VLA-4), raised much attention due to its being constitutively expressed on the surface of lymphocytes and most leukocytes, and being involved in coordinating leukocyte homing in various tissues [6].

As a fruitful consequence of intense investigation on integrins, several integrin antagonists have been validated as drugs. For example, diverse small molecules and antibodies, including eptifibatide, tirofiban, and abciximab, which target the platelet-specific integrin α_IIb_β_3_, are effectively used as therapeutic agents in the treatment of acute coronary syndromes and prevention of myocardial infarct following coronary intervention [7]. On the other hand, the known roles of leukocyte-specific integrins in events such as inflammation and host defense has prompted parallel anti-integrin strategies, yielding effective therapeutic anti-inflammatory agents [8,9]. Indeed, targeting α_4_ integrins has proven to be effective for the treatment of inflammatory diseases, including multiple sclerosis and Crohn’s disease, with some monoclonal antibodies being approved for clinical practice [10,11].

Inflammatory responses are crucial for host defense and are subjected to a complex system of control, aiming to prevent tissue damage and dangerous consequences. Since many inflammatory diseases are characterized by an influx of lymphocytes and leukocytes in the inflamed tissue, there is a keen interest in finding and testing compounds that have the potential to modulate these processes [12]. In this context, integrin activation during the different steps of the leukocyte adhesion cascade is the result of a fine-tuned orchestra of activation pathways and local regulatory networks at the site of inflammation, whose malfunctioning may cause severe disease patterns. The diseases associated with α_4_β_1_ (and α_4_β_7_) integrins are mainly of inflammatory and autoimmune nature, implying a pathological accumulation of activated leukocytes in the affected tissues such as, for example, inflammatory bowel disease, Crohn’s disease, rheumatoid arthritis, asthma, multiple sclerosis, dry eye disease and allergic conjunctivitis [10,13]. Moreover, the strict correlation between inflammation and cancer is well established at present, and immunomodulation is recognized as a useful tool not only in the treatment of inflammatory and autoimmune pathologies, but also as an adjuvant in tumor therapy. It is known that chronic inflammatory states and tumor development are closely related and mutually supportive [14]. Indeed, during chronic inflammation, the release of chemokines and growth factors supports tumor development, while, on the other hand, the tumor state can induce the upregulation of immunosuppressive molecules and the dysregulated T-cell-mediated host responses. In addition, the α_4_β_1_ integrin was demonstrated to play a pivotal role in tumor angiogenesis associated with chronic inflammation, a condition that may promote the angiogenetic switch in tumors [15]. Integrin α_4_β_1_ is also involved in the recruitment of progenitor cells (multipotent cells derived from bone marrow stem cells), in the transendothelial tumor cell migration and, due to its overexpression in melanoma cells, α_4_β_1_ is also considered a marker of metastatic risk [16,17].

In this complex scenario, the possibility to interfere with integrin activity is of great interest and α_4_ integrins have become a target for fine modulation by interaction with small-molecule ligands, based on the emerging idea that *antagonist* ligands may interfere in leukocyte primary functions while, on the other hand, *agonist* ligands can serve to promote some useful integrin functions. Enhancement of cell adhesion, for example, impairing cell detachment, may prevent tumor cell migration and metastasis processes, or may induce progenitor cell retention for stem cell therapy [18].

**Figure 1:**
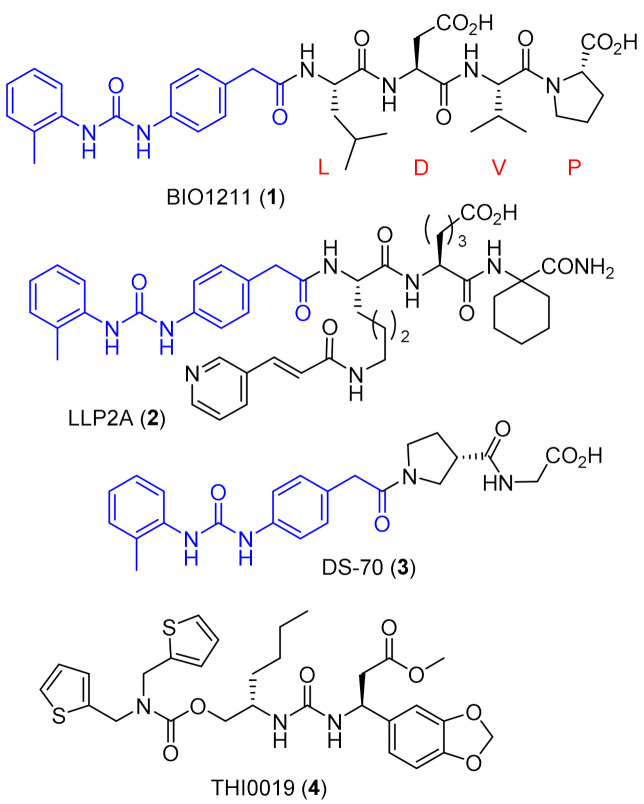
Examples of linear peptidomimetics targeting the α_4_β_1_ integrin receptor, some of which have been advanced in preclinical studies [13,18,22,24]. The MPUPA moiety is depicted in blue.

The natural ligands of α_4_ integrins comprise the vascular adhesion molecule-1 (VCAM-1) and the alternatively spliced connecting segment 1 (CS1) region of fibronectin (FN). In particular, FN is recognized through the Leu–Asp–Val (LDV) binding epitope [19], while VCAM-1 interacts with its receptor via the homologous and essentially isosteric binding sequence Gln–Ile–Asp–Ser–Pro–Leu (QIDSPL) [20]. The discovery that these short amino acidic sequences are minimal recognition motifs has prompted the research of small-molecule peptidomimetics resembling the natural binding epitope and fitting into the groove at the α and β subunit interface [10,21]. Figure 1 collects some notable results in the discovery of linear peptidomimetic ligands, reminiscent of the LDV sequence and targeting the α_4_β_1_ receptor.

In 1999, the Adams’ research group reported the synthesis of BIO1211 (compound **1**, Figure 1) [22], a potent and selective α_4_β_1_ antagonist, which was shown to inhibit the α_4_β_1_/VCAM-1 interaction with an IC_50_ of 4 nM (Jurkat cell adhesion assay) and to possess a marked selectivity for α_4_β_1_ as compared to α_4_β_7_ integrin (IC_50_ α_4_β_7_ = 2 µM).

BIO1211 is based on the peptide sequence Leu–Asp–Val–Pro (LDVP) substituted at the amino terminus with the 4-[(*N*-2-MethylPhenyl)Ureido]PhenylAcetyl group (MPUPA). The introduction of this last moiety was demonstrated to produce a substantial increase in both potency and enzymatic stability as compared to the LDV peptide precursor [23]; for this reason, BIO1211 is commonly used as a reference compound in many studies aiming to developing new α_4_β_1_ ligands. The in vitro efficacy and potency of this compound were also confirmed in vivo: when administered as an aerosol, it showed prophylactic efficacy in a sheep model of allergic bronchoconstriction, electing this nonsteroidal compound as the first small-molecule α_4_β_1_ antagonist to enter clinical trials. However, the residual peptide nature of BIO1211 caused a certain enzymatic instability. To overcome this behavior, a number of bioactive peptidomimetics have been prepared (Figure 1), which share common structural features, including an aromatic cap at the *N*-terminus, a suitable spacer, and a carboxylic group mimetic of the Asp residue, with BIO1211 [10]. Compound LLP2A (**2**, Figure 1), proposed by Peng et al. in 2006 [24], was identified in a competitive cell-based screening under a high concentration of soluble BIO1211. It showed an exceptionally high affinity toward α_4_β_1_ receptor (IC_50_ = 2 pM, Jurkat cell assay) without any effect on the cell proliferation and survival of α_4_β_1_–positive cells. For this reason, it was differently functionalized with NIR-fluorescence probes, or labelled with radionuclides (^111^In, ^64^Cu, ^99m^Tc and ^18^F) to image several different tumors, including melanoma [25,26]. Recently, due to its high binding affinity to integrin α_4_β_1_, which is highly expressed on mesenchymal stem cells (MSCs) and regulates MSC homing, adhesion, migration and differentiation, LLP2A has also been exploited for tissue engineering and regenerative medicine applications [27].

According to a common trend in bioactive peptide research, the introduction of cyclic scaffolds, including proline derivatives and other five-membered heterocycles, has been exploited by many researchers as amide bond isosteres and conformational restraints in the design and synthesis of peptidomimetic integrin ligands [28]. Along this line, the insertion of a D-configured β2-proline scaffold into a peptidomimetic structure led to the development of compound DS-70 (**3**, Figure 1), which demonstrated a high binding affinity for human α_4_β_1_ integrin and potent antagonist activity of α_4_–mediated cell adhesion. Additionally, it was successfully tested in a guinea pig preclinical model of allergic conjunctivitis [13]. Lastly, compound THI0019 (**4**, Figure 1) [18] was the first α_4_β_1_ agonist designed and synthesized starting from a potent α_4_β_1_ antagonist as a template [29]. THI0019 was generated by introducing two structural modifications into a previously identified α_4_β_1_ antagonist. As a result, THI0019 enhanced the rolling, spreading, adhesion, and migration of endothelial progenitor cells in vitro in a α_4_β_1_-dependent fashion; the authors suggested that compound **4** could temporarily occupy the ligand binding pocket, inducing a small conformational change in the receptor that favors agonist displacement and binding of natural ligand, thus opening opportunities for stem cell therapy [18].

Despite the relevant results obtained in the preclinical evaluation of these molecules as targeting motifs in the construction of imaging probes, potential treatments in ocular diseases, or innovative materials for regenerative medicine, there is still ample room for the development of new and structurally varied binders, which may enrich the pool of existing α_4_β_1_ ligands.

**Figure 2:**
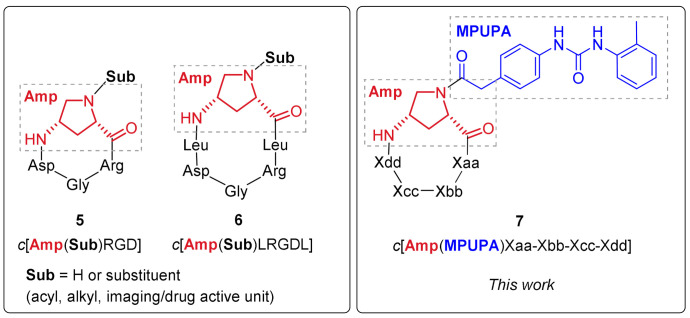
Amp-based cyclopeptides directed to RGD-recognizing α_V_β_3_, α_V_β_5_, and α_V_β_6_ integrins (compounds **5** and **6**) [30,31,32,33,34,35,36,37,38,39,40], and general structure of cyclopeptidomimetics **7** designed and synthesized to target α_4_β_1_ integrin in the present study.

In recent years, the exploitation of the *cis*-4-amino-L-proline residue (Amp) as a conformation-inducing scaffold led to the development of novel classes of RGD-based cyclopeptide ligands of type **5** and **6** (Figure 2) targeting α_V_β_3_, α_V_β_5_, and/or α_V_β_6_ integrin receptors with a good-to-high-affinity and selectivity [30,31,32]. These integrins are known to be directly involved in the evolution and diffusion of metastatic tumor cells and angiogenesis, as well as in the development of organ fibrosis.

The Amp scaffold is a new-to-nature, yet nature-reminiscent small-molecular entity, which can be grafted onto the peptide sequence of interest and impart proper ligand conformation [30], while conferring stability toward enzymatic degradation. Moreover, the Amp nucleus possesses a *N*^α^-proline site free for covalent bonding to useful functional units; indeed, the Amp-based cyclopeptide cores were covalently conjugated to either fluorescent tags, chelating units, or established therapeutic drugs to obtain hybrid dual-active structures and nanoparticles [33,34,35,36,37,38,39,40].

In the present study, the Amp scaffold was selected as the core unit for building up a new class of cyclic small-molecule peptidomimetics by linking it to proper pharmacophoric groups, aiming to target the α_4_β_1_ integrin receptor. To explore this possibility, we designed and synthesized a small collection of cyclic aminoproline-based peptidomimetics of general formula *c*[Amp(MPUPA)Xaa-Xbb-Xcc-Xdd] **7** (Figure 2), in which the Amp scaffold was grafted onto suitable peptide sequences (LDV motif and analogues) and functionalized at the *N*^α^-proline site with the well-known α_4_-integrin targeting MPUPA moiety.

In this work, we report the molecular modelling-driven design, the synthesis, and the chemical characterization of a collection of seven tetra- and pentacyclopeptidomimetics of type **7**, as well as the evaluation of their binding competence towards the α_4_β_1_ integrin receptor by cell adhesion assays using Jurkat cells in the presence of VCAM-1, with the aim to preliminarily assess their ability to bind α_4_β_1_ integrin and possibly serve as modulators of integrin function.

## 2. Results 

### 2.1. Design of Novel α_4_β_1_ Ligands

The study of the interactions between ligands and their biological targets greatly benefits from the availability of ligand-receptor crystallographic insights; since no X-ray analyses exist to date on the crystal structure of the α_4_β_1_ receptor, or of the same receptor in complex with its small-molecule ligands, the design of a new class of cyclic Amp-based peptidomimetics required the generation and validation of a α_4_β_1_ receptor model by molecular modelling studies [41].

**Figure 3:**
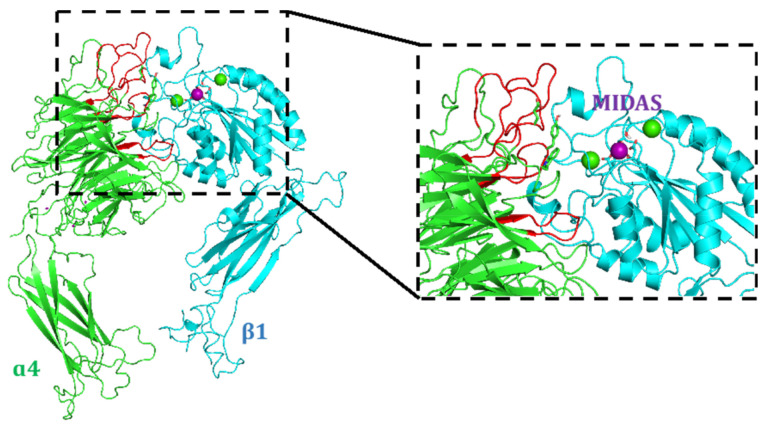
The developed α_4_β_1_ complex shows the classical structural organization of the integrin family; α_4_ subunit (green cartoon), β_1_ subunit (cyan cartoon); the Ca^2+^ and Mg^2+^ ions of MIDAS are depicted as green and purple spheres, respectively. The portion of the β-propeller domain (α_4_ subunit) involved in ligand binding region is evidenced in red.

The work started from the atomic coordinates of the single α_4_ and β_1_ domains, which were available from the α_4_β_7_ integrin complex (PDB code: 3V4V) [42] and the α_5_β_1_ integrin complex (PDB code: 3VI4) [43], respectively. In fact, these integrins possess a high degree of structural conservation, a large, solvent-exposed ligand-binding site at the α/β interface, and a divalent cation (Mg^2+^) at the metal ion-dependent adhesion site (MIDAS), which may be involved in a coordinated bond with a carboxylate group of the ligand. Using the α_5_β_1_ integrin complex as a template, the α_4_ subunit was aligned with α_5_ bound to β_1_; then, the α_5_ subunit was removed, giving a preliminary α_4_β_1_ complex. The integrin complex thus obtained was refined and optimized by a minimization protocol (Figure 3) and then subjected to a validation procedure through docking studies.

**Figure 4:**
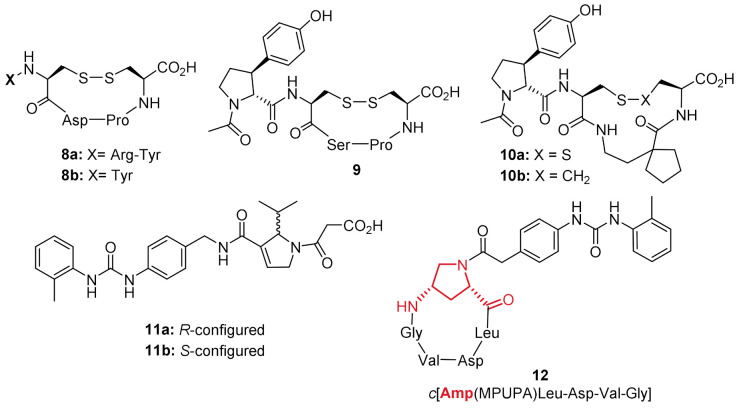
Known cyclic peptides (**8a**, **8b**, and **9**), their spirocyclic analogues (**10a**, **10b**), the dehydro-β-proline peptidomimetics **11a** and **11b**, and the novel Amp-based cyclopentapeptidomimetic **12** used for docking studies in this work.

To this aim, eight known α_4_β_1_ integrin antagonists were selected, namely compound BIO1211 (**1**, Figure 1), compounds **8a**, **8b**, **9**, **10a**, **10b**, **11a**, and **11b** (Figure 4), along with one novel Amp-based cyclic candidate (compound *c*[Amp(MPUPA)-Leu-Asp-Val-Gly] **12**, Figure 4). This small collection of known peptidic and peptidomimetic structures showed a certain level of molecular diversity and inhibitory potencies towards α_4_β_1_ integrin, ranging from micromolar to low nanomolar values [44,45,46].

In particular, the low-nanomolar cyclic peptides **8a**, **8b**, and **9**, containing the Cys-Asp-Pro-Cys or Cys-Ser-Pro-Cys core structures, and their spirocyclic analogues **10a** and **10b**, constrained by a disulfide (or a thioether) bridge, were conceived to mimic the essential α_4_β_1_ IDS or LDV binding sequences [44,45]. Compound **11a** represents one of the linear analogues of BIO1211 obtained by a retro-sequence strategy and containing a dehydro-β-proline ring which, similarly to compounds **8**–**10**, showed a potent inhibitory activity of α_4_β_1_/VCAM interaction with IC_50_ in the nanomolar range [46], accompanied by a superior enzymatic stability respect to cyclic peptides **8**–**10**.

**Figure 5:**
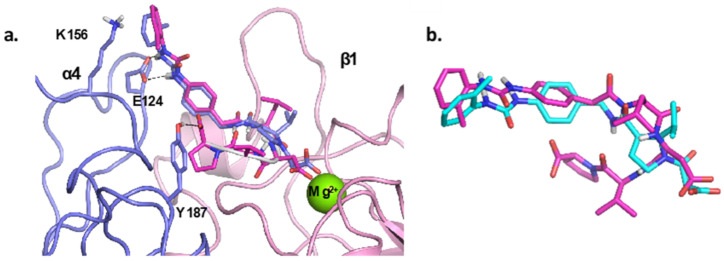
(**a**) Binding poses of BIO1211 (purple sticks) and **11a** (blue sticks) in the binding site of α_4_β_1_ integrin (α_4_ subunit blue ribbon; β_1_ subunit pink ribbon). The Mg^2+^ cation is reported as a green sphere. (**b**) Overlapping of compounds BIO1211 (purple sticks) and **11a** (cyan sticks) obtained by docking studies.

Figure 5 shows the binding poses of compounds BIO1211 and **11a** within the α_4_β_1_ binding site, as well as their overlapping structures. The analysis of these binding poses revealed that both compounds can interact with Mg^2+^ cation in the β subunit and share a similar disposition within the binding pocket of the receptor, with the common functional groups interacting with the same amino acid residues in the α subunit. Notably: (i) the ureido group within MPUPA of both compounds establishes a bidentate interaction with Glu124 (E124) residue, (ii) the terminal aromatic ring of the ureido group establishes a cation-π interaction with Lys156 (K156) residue, (iii) the isopropyl group of **11a** adopts a spatial orientation similar to the leucine side chain of BIO1211. This last observation would explain the experimental evidence showing that compound **11b** (the enantiomer of **11a**) is considerably less active on α_4_β_1_ [46]. Furthermore, BIO1211 establishes a H-bond with Tyr187 (Y187), a crucial interaction, as highlighted by reported mutagenesis studies [47]. The rationalization of the binding poses of the selected compounds proved to be in agreement with the SAR studies reported in the literature [44,46], supporting the reliability of the developed receptor model.

**Figure 6:**
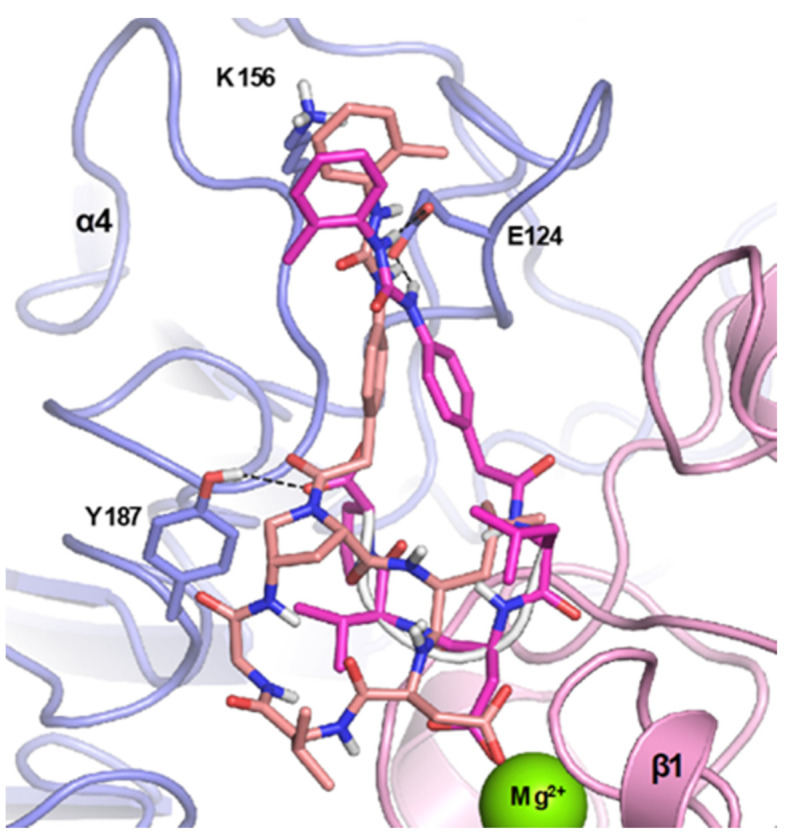
Binding poses of Amp-cyclopeptide **12** (pink sticks) and BIO1211 (purple sticks) in the binding site of α_4_β_1_ integrin (α_4_ subunit blue ribbon; β_1_ subunit pink ribbon).

The validated model was used in the subsequent docking studies, where the same experimental protocol was applied, to identify the binding modes of Amp-bearing α_4_β_1_-ligands, and to predict possible structural modifications improving affinity toward the α_4_β_1_ integrin. To this end, the docking procedure was used to evaluate *c*[Amp(MPUPA)Leu-Asp-Val-Gly] (**12**) as a new potential α_4_β_1_ ligand. In Figure 6, the binding pose of compound **12** is shown and compared to that of BIO1211.

From the analysis of the docking poses, we noticed that cyclopeptide **12** (pink sticks) would be able to establish some comparable interactions to BIO1211 (purple sticks) in the binding pocket of the α_4_β_1_ receptor model. Compound **12** seems to be particularly able to (i) chelate the divalent cation (Mg^2+^) through the carboxylate group of the Asp residue, (ii) interact with the amino acidic residues Tyr187, Lys156 and Glu124 in a similar way as BIO1211; (iii) its Val residue seems to assume the favorable spatial orientation that was observed for BIO1211, and (iv) the MPUPA moiety of both compounds occupies the same region.

**Figure 7:**
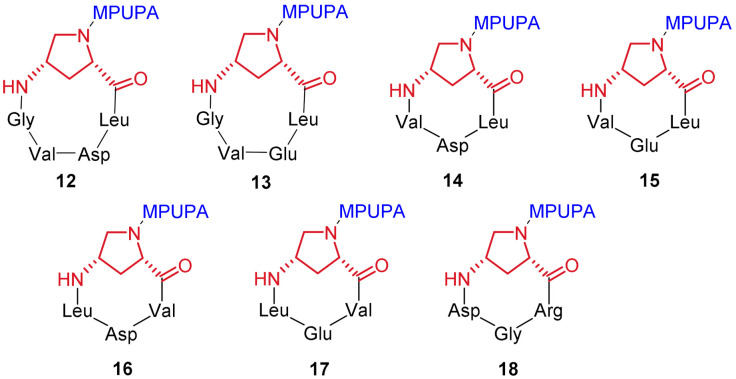
The collection of Amp-MPUPA-bearing cyclopeptidomimetics **12**–**18** designed and synthesized in this study.

Starting from compound **12**, six additional cyclic Amp-based cyclopeptide derivatives were designed, namely, compounds **13**–**18** (Figure 7), to be launched in the synthesis program. The design was rooted in the following considerations: (i) substitution of the Glu residue for Asp could further favor the interaction of the carboxylate group of the side chain with the divalent cation of MIDAS (e.g., compound **12** vs. **13**, **14** vs. **15**, **16** vs. **17**), (ii) restriction of the cyclopeptide ring via Gly depletion could provide insights about the influence of ring size and constrain on binding affinity (e.g., pentapeptide compounds **12–13** vs. tetrapeptide analogues **14**–**18**); (iii) exploitation of retro-sequences could expand exploration of the pharmacophoric space (e.g., VDL-based compound **16** vs. LDV counterpart **14**, and VEL-based compound **17** vs. LEV counterpart **15**), (iv) substitution of the RGD sequence for LDV would generate derivative **18**, which could likely be used as a negative control in α_4_β_1_-directed biological assays.

### 2.2. Synthesis of Novel α_4_β_1_ Ligands

**Scheme 1:**
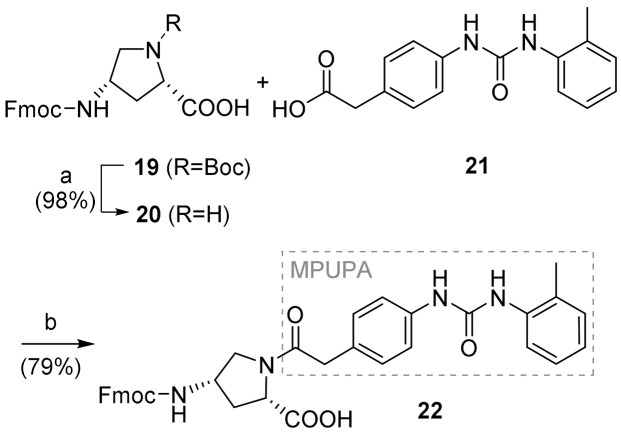
Synthesis of *N*-Fmoc-Amp(MPUPA)-OH scaffold **22**. Reagents and conditions: (**a**) TFA, dry DCM, rt; (**b**) HATU, HOAt, collidine, dry DMF.

The synthesis of the designed compounds **12**–**18** began with the chemoselective in-solution *N*^α^ deprotection of commercially available Fmoc-4-amino-1-Boc-pyrrolidine-2-carboxylic acid (**19**) to **20** (Scheme 1) and subsequent functionalization with the 4-[(*N*-2-methylphenyl)ureido]phenylacetyl (MPUPA) moiety **21**, to provide the *N*-Fmoc-Amp(MPUPA)-OH scaffold **22** (79% yield) to be used in the following SPPS procedures. The MPUPA moiety **21** was instead synthesized, with good yield, starting from the commercially available precursors, *o*-tolyl isocyanate and 4-aminophenylacetic acid, following literature procedure [22]. The synthesis of compound **22** entailed the preliminary activation of the carboxylic function within MPUPA unit **21** by means of HATU/HOAt/collidine coupling system in dry DMF, followed by the addition of **20**.

**Scheme 2:**
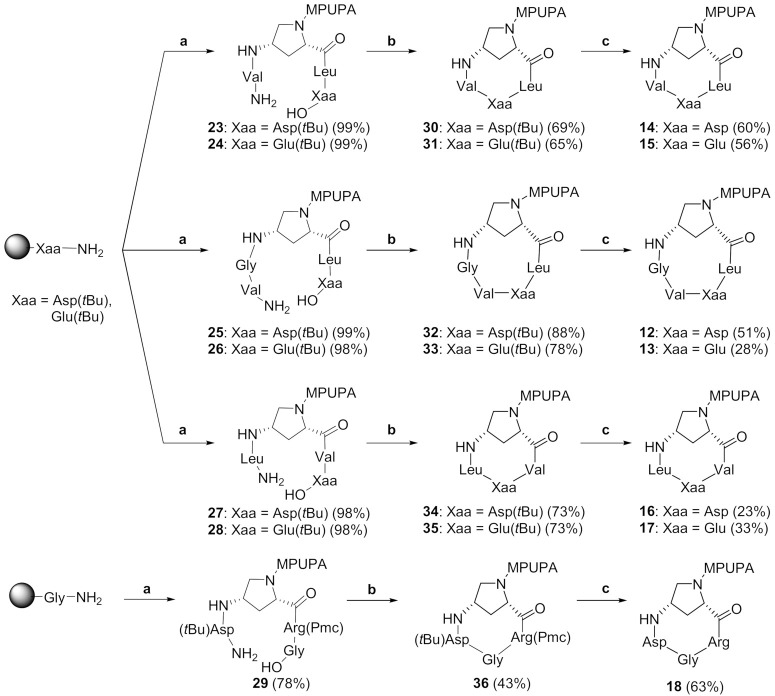
Synthesis of Amp(MPUPA)-based target compounds **12**–**18**. Reagents and conditions: (**a**) Fmoc-SPPS strategy. *Coupling*: Fmoc-amino acid, HATU, HOAt, collidine, DMF, rt. *Fmoc cleavage*: 20% piperidine in DMF, rt. *Sequence of addition to obtain compounds*: (**23**, **24**) Fmoc-Leu-OH, Fmoc-Amp(MPUPA)-OH (**22**), Fmoc-Val-OH; (**25**, **26**) Fmoc-Leu-OH, Fmoc-Amp(MPUPA)-OH (**22**), Fmoc-Gly-OH, Fmoc-Val-OH; (**27**, **28**) Fmoc-Val-OH, Fmoc-Amp(MPUPA)-OH (**22**), Fmoc-Leu-OH; (**29**) Fmoc-Arg(Pmc)-OH, Fmoc-Amp(MPUPA)-OH (**22**), Fmoc-Asp(*t*Bu)-OH. *Resin cleavage:* AcOH/TFE/DCM (1:1:3), rt. (**b**) Cyclization: HATU, HOAt, collidine, DCM/DMF (15:1), 1–3 mM, rt. (**c**) Deprotection: TFA/TIS/H_2_O (95:2.5:2.5), rt.

For the synthesis of the linear precursors of targeted cyclopeptides **12**–**18**, the Fmoc-based SPPS strategy was adopted, followed by in-solution cyclization and deprotection protocols (Scheme 2). All the linear peptide sequences were prepared, starting from the proper acid-labile chlorotrityl chloride resin preloaded with one of the three different starting amino acid residues, Asp(*t*Bu), Glu(*t*Bu) or Gly. Within all the designed peptide sequences, the aminoproline scaffold played a critical role; in fact, in all instances, this unit was in a central position within the linear peptides, creating a local constraint that would likely pre-organize the terminal chains toward the final macrocyclization step. The synthesis of the designed sequences required the stepwise addition of Fmoc-protected amino acids to the growing peptides, with alternating coupling steps (in the presence of HATU/HOAt/collidine) and Fmoc-removal procedures (by using 20% piperidine/DMF solution); then, the linear peptide sequences were readily cleaved from the resin using the conventional AcOH/TFE/DCM mixture. The crude linear peptides **23**–**29** were obtained in yields ranging from 78% to 99% for the entire solid phase sequences.

The linear peptides **23**–**29** were then subjected to delicate, in-solution, head-to-tail cyclization. The cyclization reactions were carried out under diluted conditions (1–3 mM) in a solution of dry DCM/DMF solvent mixture, in a 15:1 ratio. The crude cyclized peptides were purified by automated flash chromatography, furnishing the protected cyclized peptides **30**–**36** with yields ranging from 43% to 88%. Finally, side-chain deprotection of the cyclic peptides was carried out under acidic conditions (TFA/TIS/H_2_O 95:2.5:2.5). Compounds **12**–**18** were recovered as TFA salts after RP-HPLC purification, in yields ranging from 23% to 63%, with overall yields ranging from 21% to 44%. Target compounds **12**–**18** were fully characterized by high-resolution ESI mass spectrometry as well as various NMR techniques.

### 2.3. Biological Evaluation

To investigate the ability of the newly synthesized cyclopeptidomimetics **12**–**18** to recognize and bind α_4_β_1_ integrin, cell adhesion assays were performed on VCAM-1. The compounds were evaluated for their ability to interfere with α_4_β_1_ integrin-mediated cell adhesion by using Jurkat cells, which are known to constitutively express this integrin [46,48,49,50,51]. Compound BIO1211 (**1**) was included as a reference antagonist ligand, which is able to significantly reduce Jurkat cell adhesion to VCAM-1. The results of cell adhesion assays are summarized in Table 1.

Under the adopted experimental conditions, most of the synthesized compounds were unable to compete with VCAM-1 for the binding to the α_4_β_1_ receptor expressed on Jurkat cells and no effect was detected on the impairing or promoting of cell adhesion (anti-adhesive or pro-adhesive effect) at the tested concentrations (ranging from 0.1 nM to 100 μM). The new cyclopeptidomimetic **16**, instead, was able to modulate α_4_β_1_ integrin-mediated cell adhesion, with an interesting potency in the submicromolar range; in particular, it behaved as an agonist, as it was able to increase Jurkat cell adhesion to VCAM-1 as compared to the control. More specifically, this compound, which features a constrained cyclotetrapeptide ring containing the retro-sequence Val-Asp-Leu, showed a dose-dependent enhancement in cell adhesion with an EC_50_ of 0.37 μM, and, for this reason, it was referred to as an agonist.

**Figure 8:**
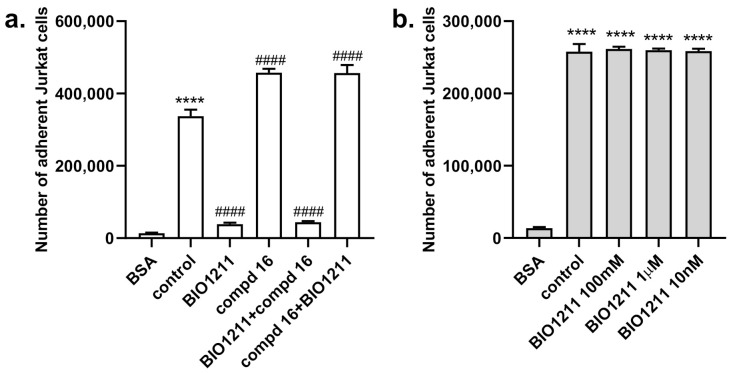
Jurkat cell adhesion to wells coated with VCAM-1 [2 μg/mL; panel (**a**)] or compound **16** [10 μg/mL; panel (**b**)] to evaluate any possible ligand binding competition. (**a**)**.** The antagonist BIO1211 (1 μM) significantly reduced Jurkat cell adhesion to VCAM-1; on the contrary compound **16** (100 μM) behaved as an α_4_β_1_ integrin agonist, increasing the adhesion of Jurkat cells to VCAM-1. When Jurkat cells were pre-incubated with BIO1211 and then with compound **16**, the latter was not able to modify the reduction in adhesion induced by BIO1211. Similarly, BIO1211 was not able to revert the effect induced by pre-incubation with compound **16**. (**b**). Even in absence of VCAM-1, compound **16** was able to induce Jurkat cell adhesion; BIO1211 (10 nM–100 mM) was not able to reduce the increment of cell adhesion induced by compound **16**. Control cells were not pre-incubated with any compound. Jurkat cells plated in wells coated with 10 μg/mL bovine serum albumin (BSA) were considered as negative control. Each value is the mean ± SD from four separate experiments carried out in quadruplicate. **** *p* < 0.0001 vs. BSA-coated wells; ^####^ *p* < 0.0001 vs. control (Newman–Keuls test after ANOVA).

In an attempt to rationalize our results, two additional experiments were envisaged to evaluate the possible competition for VCAM-1 binding site between Amp-based cyclotetrapeptide **16** and BIO1211, which is described as a potent noncovalent antagonist of α_4_β_1_/VCAM-1 interaction in both receptor-binding studies and cell adhesion assays [22]. In the first set of experiments, Jurkat cells were pre-incubated with BIO1211 (1 μM) for 30 min and then incubated with compound **16** (100 μM), before being plated in VCAM-1 coated wells. As expected, BIO1211, acting as an antagonist, significantly decreased Jurkat cell adhesion to VCAM-1. Moreover, compound **16** was not able to modify the reduced cell adhesion induced by BIO1211 (Figure 8a). Similarly, when Jurkat cells were pre-incubated with compound **16**, BIO1211 did not modify the increased cell adhesion. 

In a second set of experiments, the ability of compound **16** to increase cell adhesion was tested in the absence of VCAM-1. Wells were coated by passive adsorption with compound **16** or BSA (both at 10 µg/mL) as a negative control and Jurkat cell adhesion was measured (Figure 8b). Compound **16** produced a significant adhesion of Jurkat cells, even in the presence of different concentrations of BIO1211.

With the present data, a more detailed analysis of the structure–activity relationship within our ligand collection would be unwise. 

## 3. Discussion

Two main aspects emerge from the experimental results given above, which may deserve comment: first, a discrepancy was observed between the computational-driven design and the experimental results, and second, an agonist behavior emerged in one candidate instead of the “expected” antagonist activity of the modeled structures.

Regarding the first point, it has to be underlined that, lacking sound structural details of the α_4_β_1_ integrin, a reliable model for the design of potential α_4_β_1_ ligands remains elusive, although several molecular modeling studies, computational screenings and 3D models have been reported to date [52,53,54,55]. Additionally, the high degree of conformational flexibility featuring the targeted receptor was not considered in this study, and this could have played a decisive role in decreasing the predictability potential of the molecular modeling studies.

The in vitro biological evaluation showed that, among the seven candidates, compound *c*[Amp(MPUPA)Val-Asp-Leu] (**16**) exhibited a low-micromolar (0.37 μM) agonist activity in Jurkat cell adhesion assay. The fact that the rational design based on antagonist ligands consigned an agonist product must not come as a surprise, as this was testified by notable precedents even in the field of α_4_β_1_ ligands [18,24]. It has been demonstrated that even small structural variations in the integrin ligand core can cause the shift from antagonist to agonist behavior [18,49]. 

Competition experiments involving compound **16** and BIO1211 revealed that the respective agonist and antagonist behavior were not reciprocally modified. These results seem to exclude the competition between compound **16** and BIO1211 for the same binding site, and the agonist activity of **16** could be ascribed to an interaction of this compound in a different region of the receptor. This behavior has already been observed for other ligands of this integrin; for example, for known compound LLP2A (**2**, Figure 1), whose binding site on α_4_β_1_ integrin receptor was claimed to be different, and close to (or only partially overlapping) with the binding site of VCAM-1 [24].

Finally, in contrast to RGD-dependent integrins, the binding regions of α_4_ integrins (in particular the α_4_β_7_ binding site) have been described as long and wide crevices, open at both ends and capable of the lengthwise accommodation of differently shaped binders [42]. This fact could explain that compounds **16** and BIO1211 do not seem to share the same binding region and could provide a reason for the difficulty encountered in the rational design.

## 4. Materials and Methods

### 4.1. Docking Studies

#### 4.1.1. Protein Setup

α_5_β_1_ (PDB code: 3VI4) [43] and α_4_β_7_ (PDB code: 3V4V) [42] crystal structures were used for generation of the α_4_β_1_ complex: the α_4_ subunit was obtained by the α_4_β_7_ complex, while the β_1_ subunit was derived from α_5_β_1_ receptor. Using the α_5_β_1_ integrin complex as a template, the α_4_ subunit was aligned with α_5_ bound to β_1_ (α_5_β_1_), then the α_5_ subunit was removed, giving a preliminary α_4_β_1_ complex. The complex was prepared by using the Protein Preparation Wizard tool of Maestro 9.1 (https://www.schrodinger.com/; accessed on 9 November 2018) minimized by a multi-step protocol in which the harmonic restraints were gradually scaled. The complex obtained was used for the following docking studies. 

#### 4.1.2. Ligand Docking Calculations

All docking studies were carried out using the same experimental protocol. The structures of the different antagonists were prepared from the fragment-building tool available in Maestro 9.1 and the geometries were optimized using the force field OPLS-2005 [56]. The docking grid was centered on the Mg^2+^ atom and a grid size of 12 × 12 × 12 Å was used. Docking studies were performed using Glide as software, the SP method and the enhanced sampling method for conformational exploration of the different ligands. The remaining docking parameters were used as default.

### 4.2. Chemistry

#### General Information

H-Gly-2-ClTrt resin (loading 0.63 mmol/g), H-Asp(*t*Bu)-2-ClTrt resin (loading 0.74 mmol/g), H-Glu(*t*Bu)-2-ClTrt resin (loading 0.85 mmol/g) were purchased from Novabiochem, (2*S*,4*S*)-Fmoc-4-amino-1-Boc-pyrrolidine-2-carboxylic acid from PolyPeptide and all other reagents from Alfa Aesar, TCI and Sigma-Aldrich. Automated flash column chromatography was carried out with the Biotage Isolera One system using Biotage KP-C18-HS (reverse phase). ESI-mass spectra were recorded on UHPLC/ESI-MS system (ACQUITY Ultra Performance LC; ESI, positive ions, Single Quadrupole analyzer) and are reported in the form of (*m*/*z*). HPLC purifications were performed on a Prostar 210 apparatus (Varian, UV detection) equipped with C_18_-10 µm column (Discovery BIO Wide Pore 10 × 250 mm or 21.2 × 250 mm). Routine NMR spectra were recorded on Avance 300 or 400 (Bruker) NMR spectrometers. Chemical shifts (δ) are reported in parts per million (ppm) with CD_2_HOD resonance peak set at 3.31 ppm. Multiplicities are indicated as s (singlet), d (doublet), t (triplet), q (quartet), m (multiplet), and b (broad). Coupling constants, *J*, are reported in Hertz. ^1^H NMR assignments are corroborated by 1D and 2D experiments (gCOSY sequences). Optical rotations were measured using a Perkin–Elmer model 341 polarimeter at ambient temperature using a 100 mm cell with a 1 mL capacity and are given in units of 10^−1^ deg cm^2^ g^−1^. High resolution mass analysis (ESI) was performed on LTQ ORBITRAP XL Thermo apparatus and are reported in the form of (*m*/*z*). (2*S*,4*S*)-4-*N*-(9-Fluorenylmethoxycarbonyl)aminoproline (**20**) and 4-[[[(2-methylphenyl)amino] carbonyl]amino]phenylacetic acid (MPUPA-OH) (**21**) were prepared according to the literature procedures [22,32].

### 4.3. Experimental Synthetic Procedures and Characterization Data

#### 4.3.1. (2*S*,4*S*)-1-(MPUPA)-4-(Fmoc)aminoproline [Fmoc-(MPUPA)Amp-OH] (**22**)

To a stirred solution of MPUPA-OH **21** (107 mg, 0.38 mmol, 1.1 equiv), HATU (144 mg, 0.38 mmol, 1.1 equiv) and HOAt (51 mg, 0.38 mmol, 1.1 equiv) in dry DMF (2 mL), 2,4,6-collidine (95 µL, 0.72 mmol, 2.1 equiv) was added and the system left to stir for 30 min, under argon at room temperature. A solution of compound **20** and 2,4,6-collidine (46 µL, 0.34 mmol, 1 equiv) in dry DMF (8 mL) was then added dropwise to the reaction mixture, over 20 min. The reaction reached completion in 40 min and was then treated with an aqueous solution of HCl (0.1 N) to precipitate the product. The crude residue was filtered and purified by reverse phase flash chromatography [H_2_O (0.1% TFA)/MeCN: linear gradient 80:20 to 20:80] furnishing compound **22** as withe glassy solid (168.2 mg, yield 79%). ^1^H NMR (MeOD, 400 MHz): 7.76 (d, *J* = 7.5 Hz, 2H, ArH Fmoc), 7.66–7.56 (m, 3H, ArH Fmoc and MPUPA), 7.42–7.33 (m, 4H, ArH Fmoc and MPUPA), 7.32–7.24 (bm, 2H, ArH Fmoc), 7.23–7.11 (m, 4H, ArH MPUPA), 7.01 (ddd, *J* = 7.4, 7.4, 1.0 Hz, 1H, H6′ MPUPA), 4.40 (t, *J* = 6.8 Hz, 1H, H2 Amp), 4.31 (m, 2H, H1″ Fmoc), 4.24–4.14 (bm, 2H, H2″ Fmoc and H4 Amp), 3.94–3.86 (m, 1H, H5 Amp), 3.66 (d, *J* = 15.9 Hz, 1H, H1′ MPUPA), 3.65 (d, *J* = 15.8 Hz, 1H, H1′ MPUPA), 3.35 (m, 1H, H5 Amp), 2.60–2.49 (bm, 1H, H3 Amp), 2.25 (s, 3H, H8′ MPUPA), 1.95–1.84 (bm, 1H, H3 Amp). ^13^C NMR (MeOD, 400 MHz): 174.0, 171.3, 156.6, 154.4, 143.8, 141.2, 138.1, 136.5, 130.1, 129.7, 129.2, 128.3, 127.4, 126.8, 126.1, 124.8, 124.0, 123.0, 119.6, 119.0, 66.4, 57.8, 52.1, 51.3, 50.2, 40.4, 34.2, 16.7.

#### 4.3.2. General Procedure for Fmoc-Based SPPS

Linear peptides **23**–**29** were prepared according to the following general procedure, using the preloaded resins: (i) H-Asp(*t*Bu)-2-ClTrt resin (loading 0.74 mmol/g) (**23**, **25**, **27**); (ii) H-Glu(*t*Bu)-2-ClTrt resin (loading 0.85 mmol/g) (**24**, **26**, **28**); (iii) H-Gly-2-ClTrt resin (loading 0.63 mmol/g) (**29**). *Resin swelling*. The desired resin (1 equiv) was swollen in a solid phase reaction vessel with dry DMF (2 mL) under mechanical stirring; after 40 min the solvent was drained and the resin was washed with DCM (2×) and DMF (2×). *Peptide coupling*. A preformed solution of Fmoc-AA-OH (1.5 equiv) in dry DMF (2 mL) was treated with HATU (2 equiv), HOAt (2 equiv) and 2,4,6-collidine (2 equiv), and stirred for 10 min before adding to the resin. The mixture was shaken at room temperature for 5 h. Completion of the reaction was checked by the Kaiser test. The solution was drained and the resin was washed several times with DMF (2×), *i*PrOH, (2×), Et_2_O (2×), DCM (2×). The resin was then treated with 20% piperidine in DMF (2 mL) and the mixture was stirred for 30 min (*Fmoc cleavage*). The solution was drained and the resin was washed with DMF (2×), *i*PrOH, (3×), Et_2_O (2×), DCM (2×). The couplings of the further amino acids, in the proper sequence, were carried out under the same conditions. *Resin cleavage*. After coupling of the last Fmoc-AA-OH, the resin was treated with 2 mL of the cleavage mixture DCM/TFE/glacial AcOH (3:1:1) and kept under mechanical stirring for 20 min at room temperature. The solution was recovered and the resin was carefully washed with DCM (2×). This protocol was repeated twice. The combined solution was evaporated under reduced pressure affording the desired linear peptide, which was used in the following synthetic step without further purification.

#### 4.3.3. General Procedure for Cyclization Reaction

Protected cyclic peptides **30**–**36** were prepared according to the following general procedure. A solution of linear peptide (1 equiv) and 2,4,6-collidine (3 equiv) in dry DCM/DMF solvent mixture (15:1 ratio) was prepared. The mixture was stirred under argon at room temperature and added dropwise to a solution of HATU (3 equiv) and HOAt (3 equiv) in dry DCM/DMF solvent mixture (15:1 ratio). The reaction mixture was degassed by argon/vacuum cycles (3×) and left to stir under argon at room temperature for 5 h. After reaction completion, the solution was concentrated under vacuum. The crude product was purified by RP-flash chromatography [H_2_O (0.1% TFA)/MeCN: linear gradient 80:20 to 20:80] furnishing the protected cyclic peptide as a glassy solid.

#### 4.3.4. General Procedure for Deprotection Reaction

Final cyclic peptides **12**–**18** were prepared according to the following general procedure. The protected cyclic intermediate (1 equiv) was dissolved in TFA/TIS/H_2_O (95:2.5:2.5) mixture and stirred at room temperature for 1 h. Then, the solvent was evaporated, and the crude residue was thoroughly washed with Et_2_O (4×) and petroleum ether (2×). Preparative RP-HPLC purification was performed [C_18_-10 μm, 21.2 × 250 mm column, solvent A: H_2_O (0.1% TFA) and solvent B: MeCN, flow rate 8.0 mL/min; detection at 254 nm] using the following elution gradient: 0–1 min 10% B, 1–18 min 10–45% B, 18–25 min 45% B.

#### 4.3.5. H-Val-1-(MPUPA)Amp-Leu-Asp(*t*Bu)-OH (**23**)

The synthesis of linear tetrapeptide **23** was performed following the SPPS general procedure, using the preloaded H-Asp(*t*Bu)-2-ClTrt resin (60.0 mg, 0.044 mmol, 1 equiv) and the following Fmoc-amino acids: Fmoc-Leu-OH (23.0 mg, 0.06 mmol, 1.5 equiv), Fmoc-(MPUPA)Amp-OH **22** (40.1 mg, 0.06 mmol), Fmoc-Val-OH (22.0 mg, 0.06 mmol). The linear tetrapeptide **23** (34.0 mg, yield 99%) was obtained as a white glassy solid, and used in the following synthetic step without further purification. MS (ESI^+^) *m*/*z* 780.4 [M + H]^+^.

#### 4.3.6. H-Val-1-(MPUPA)Amp-Leu-Glu(*t*Bu)-OH (**24**)

The synthesis of linear tetrapeptide **24** was performed following the SPPS general procedure, using the preloaded H-Glu(*t*Bu)-2-ClTrt resin (51.1 mg, 0.043 mmol, 1 equiv) and the following Fmoc-amino acids: Fmoc-Leu-OH (23.0 mg, 0.06 mmol, 1.5 equiv), Fmoc-(MPUPA)Amp-OH **22** (40.2 mg, 0.06 mmol, 1.5 equiv), Fmoc-Val-OH (22.0 mg, 0.06 mmol, 1.5 equiv). The linear tetrapeptide **24** (34.1 mg, yield 99%) was obtained as a white glassy solid, and used in the following synthetic step without further purification. MS (ESI^+^) *m*/*z* 794.4 [M + H]^+^.

#### 4.3.7. H-Val-Gly-1-(MPUPA)Amp-Leu-Asp(*t*Bu)-OH (**25**)

The synthesis of linear tetrapeptide **25** was performed following the SPPS general procedure, using the preloaded H-Asp(*t*Bu)-2-ClTrt resin (60.1 mg, 0.044 mmol, 1 equiv) and the following Fmoc-amino acids: Fmoc-Leu-OH (23.0 mg, 0.06 mmol, 1.5 equiv), Fmoc-(MPUPA)Amp-OH **22** (40.1 mg, 0.06 mmol, 1.5 equiv), Fmoc-Gly-OH (19.1 mg, 0.06 mmol, 1.5 equiv), Fmoc-Val-OH (22.2 mg, 0.06 mmol, 1.5 equiv). The linear tetrapeptide **25** (36.0 mg, yield 99%) was obtained as a white glassy solid, and used in the following synthetic step without further purification. MS (ESI^+^) *m*/*z* 837.4 [M + H]^+^.

#### 4.3.8. H-Val-Gly-1-(MPUPA)Amp-Leu-Glu(*t*Bu)-OH (**26**)

The synthesis of linear tetrapeptide **26** was performed following the SPPS general procedure, using the preloaded H-Glu(*t*Bu)-2-ClTrt resin (51.2 mg, 0.043 mmol, 1 equiv) and the following Fmoc-amino acids: Fmoc-Leu-OH (23.1 mg, 0.06 mmol, 1.5 equiv), Fmoc-(MPUPA)Amp-OH **22** (40.0 mg, 0.06 mmol, 1.5 equiv), Fmoc-Gly-OH (19.1 mg, 0.06 mmol, 1.5 equiv), Fmoc-Val-OH (22.2 mg, 0.06 mmol, 1.5 equiv). The linear tetrapeptide **26** (36.0 mg, yield 98%) was obtained as a white glassy solid, and used in the following synthetic step without further purification. MS (ESI^+^) *m*/*z* 794.4 [M + H]^+^.

#### 4.3.9. H-Leu-1-(MPUPA)Amp-Val-Asp(*t*Bu)-OH (**27**)

The synthesis of linear tetrapeptide **27** was performed following the SPPS general procedure, using the preloaded H-Asp(*t*Bu)-2-ClTrt resin (60.0 mg, 0.044 mmol, 1 equiv) and the following Fmoc-amino acids: Fmoc-Val-OH (22.1 mg, 0.06 mmol, 1.5 equiv), Fmoc-(MPUPA)Amp-OH **22** (40.2 mg, 0.06 mmol, 1.5 equiv), Fmoc-Leu-OH (23.2 mg, 0.06 mmol, 1.5 equiv). The linear tetrapeptide **27** (33.2 mg, yield 98%) was obtained as a white glassy solid, and used in the following synthetic step without further purification. MS (ESI^+^) *m*/*z* 780.4 [M + H]^+^.

#### 4.3.10. H-Leu-1-(MPUPA)Amp-Val-Glu(*t*Bu)-OH (**28**)

The synthesis of linear tetrapeptide **28** was performed following the SPPS general procedure, using the preloaded H-Glu(*t*Bu)-2-ClTrt resin (51.0 mg, 0.043 mmol, 1 equiv) and the following Fmoc-amino acids: Fmoc-Val-OH (22.1 mg, 0.06 mmol, 1.5 equiv), Fmoc-(MPUPA)Amp-OH **22** (40.0 mg, 0.06 mmol, 1.5 equiv), Fmoc-Leu-OH (23.1 mg, 0.06 mmol, 1.5 equiv). The linear tetrapeptide **28** (34.2 mg, yield 98%) was obtained as a white glassy solid, and used in the following synthetic step without further purification. MS (ESI^+^) *m*/*z* 794.4 [M + H]^+^.

#### 4.3.11. H-Asp(*t*Bu)-1-(MPUPA)Amp-Arg(Pmc)-Gly-OH (**29**)

The synthesis of linear tetrapeptide **29** was performed following the SPPS general procedure, using the preloaded H-Gly-2-ClTrt resin (70.1 mg, 0.044 mmol, 1 equiv) and the following Fmoc-amino acids: Fmoc-Arg(Pmc)-OH (43.2 mg, 0.06 mmol, 1.5 equiv), Fmoc-(MPUPA)Amp-OH **22** (40.0 mg, 0.06 mmol, 1.5 equiv), Fmoc-Asp(*t*Bu)-OH (27.0 mg, 0.06 mmol, 1.5 equiv). The linear tetrapeptide **29** (35 mg, yield 78%) was obtained as a white glassy solid, and used in the following step without further purification. MS (ESI^+^) *m*/*z* 1047.5 [M + H]^+^.

#### 4.3.12. *c*[(MPUPA)Amp-Leu-Asp-Val-Gly] (**12**)

Compound **32** was prepared according to the cyclization general procedure. A solution of linear peptide **25** (16.9 mg, 0.020 mmol) and 2,4,6-collidine (8.0 µL, 0.061 mmol) in dry DCM/DMF solvent mixture (25.0 mL/2.0 mL) was added dropwise to a solution of HATU (23.1 mg, 0.061 mmol) and HOAt (8.3 mg, 0.061 mmol) in dry DCM/DMF solvent mixture (15.0 mL/1.0 mL). After RP-flash chromatography, the protected cyclic peptide **32** (14.6 mg, yield 88%) was obtained as a white solid. MS (ESI^+^) *m*/*z* 818.4 [M + H]^+^. Compound **12** was prepared according to the deprotection general procedure. The protected cyclic intermediate **32** (14.6 mg, 0.018 mmol) was treated with 0.89 mL of TFA/TIS/H_2_O (95:2.5:2.5) mixture, then, after RP-flash chromatography, cyclic peptide **12** (7.0 mg, yield 51%) was obtained as a yellowish glassy solid. MS (ESI^+^) *m*/*z* 763.4 [M + H]^+^. ^1^H-NMR (MeOD, 400 MHz): *δ* 7.64 (d, *J* = 8.0 Hz, 1H, H4′ MPUPA), 7.41 (d, *J* = 8.5 Hz, 2H, H3′ MPUPA), 7.21 (d, *J* = 8.6 Hz, 2H, H2′ MPUPA), 7.18 (dd, *J* = 7.9, 7.9 Hz, 1H, H5′ MPUPA), 7.13 (d, *J* = 8.5 Hz, 1H, H7′ MPUPA), 7.04 (dd, *J* = 7.3, 7.3 Hz, 1H, H6′ MPUPA), 4.70 (dd, *J* = 10.3, 2.6 Hz, 1H, H2 Amp), 4.60 (dd, *J* = 6.9, 4.8 Hz, 1H, Hα Asp), 4.60 (m, 1H, H4 Amp), 4.13 (m, 1H, Hα Gly), 4.10 (m, 1H, Hα Leu), 4.02 (m, 1H, Hα Val), 3.95 (dd, *J* = 11.2, 6.6 Hz, 1H, H5 Amp), 3.72 (d, *J* = 15.3 Hz, 1H, H1′ MPUPA), 3.69 (d, *J* = 15.6 Hz, 1H, H1′ MPUPA), 3.54 (m, 1H, Hα Gly), 3.48 (m, 1H, H5 Amp), 3.05 (dd, *J* = 17.1, 7.2 Hz, 1H, Hβ Asp), 2.95 (dd, *J* = 17.0, 4.9 Hz, 1H, Hβ Asp), 2.62 (m, 1H, H3 Amp), 2.31 (s, 3H, H8′ MPUPA), 2.24 (m, 1H, H3 Amp), 1.71 (m, 3H, Hβ Leu, Hγ Leu), 0.99 (m, 12H, CH_3_ Leu, CH_3_ Val). [α]_D_^25^: −37 (*c* 1.0, MeOH).

#### 4.3.13. *c*[(MPUPA)Amp-Leu-Glu-Val-Gly] (**13**)

Compound **33** was prepared according to the cyclization general procedure. A solution of linear peptide **26** (18.2 mg, 0.03 mmol) and 2,4,6-collidine (8.7 µL, 0.066 mmol) in dry DCM/DMF solvent mixture (26.0 mL/2.0 mL) was added dropwise to a solution of HATU (24.9 mg, 0.07 mmol) and HOAt (8.9 mg, 0.066 mmol) in dry DCM/DMF solvent mixture (16.0 mL/1.0 mL). After RP-flash chromatography, the protected cyclic peptide **33** (14.2 mg, yield 78%) was obtained as a white solid. MS (ESI^+^) *m*/*z* 833.5 [M + H]^+^. Compound **13** was prepared according to the deprotection general procedure. The protected cyclic intermediate **33** (14.2 mg, 0.017 mmol) was treated with 0.85 mL of TFA/TIS/H_2_O (95:2.5:2.5) mixture and, after RP-flash chromatography, cyclic peptide **13** (3.7 mg, yield 28%) was obtained as a white-yellow glassy solid. MS (ESI^+^) *m*/*z* 777.4 [M + H]^+^. ^1^H-NMR (MeOD, 400 MHz): *δ* 7.64 (d, *J* = 8.0 Hz, 1H, H4′ MPUPA), 7.41 (d, *J* = 8.5 Hz, 2H, H3′ MPUPA), 7.21 (d, *J* = 8.6 Hz, 2H, H2′ MPUPA), 7.18 (dd, *J* = 7.9, 7.9 Hz, 1H, H5′ MPUPA), 7.13 (d, *J* = 8.5 Hz, 1H, H7′ MPUPA), 7.04 (dd, *J* = 7.3, 7.3 Hz, 1H, H6′ MPUPA), 4.70 (dd, *J* = 10.6, 3.3 Hz, 1H, H2 Amp), 4.62 (m, 1H, H4 Amp), 4.38 (dd, *J* = 9.1, 3.8 Hz, 1H, Hα Glu), 4.21 (d, *J* = 17.0 Hz, 1H, Hα Gly), 4.16 (dd, *J* = 9.4, 5.9 Hz, 1H, Hα Leu), 4.03 (dd, *J* = 11.0, 7.3 Hz, 1H, H5 Amp), 3.76 (d, *J* = 7.0 Hz, 1H, Hα Val), 3.71 (d, *J* = 3.9 Hz, 2H, H1′ MPUPA), 3.47 (d, *J* = 17.1 Hz, 1H, Hα Gly), 3.45 (m, 1H, H5 Amp), 2.70 (ddd, *J* = 14.2, 9.4, 9.4 Hz, 1H, H3 Amp), 2.39 (m, 2H, Hγ Glu), 2.35 (m, 1H, H3 Amp), 2.31 (s, 3H, H8′ MPUPA), 2.26 (m, 1H, Hβ Glu), 2.14 (m, 1H, Hβ Val), 2.05 (m, 1H, Hβ Glu), 1.77 (m, 1H, Hγ Leu), 1.69 (m, 2H, Hβ Leu), 1.02 (m, 6H, 2CH_3_ Val), 0.97 (m, 6H, 2CH_3_ Leu). [α]_D_^25^: −38 (*c* 1.0, MeOH).

#### 4.3.14. *c*[(MPUPA)Amp-Leu-Asp-Val] (**14**)

Compound **30** was prepared according to the cyclization general procedure. A solution of linear peptide **23** (14.4 mg, 0.012 mmol) and 2,4,6-collidine (7.3 µL, 0.055 mmol) in dry DCM/DMF solvent mixture (10.0 mL/1.0 mL) was added dropwise to a solution of HATU (21.1 mg, 0.055 mmol) and HOAt (7.5 mg, 0.055 mmol) in dry DCM/DMF solvent mixture (22.4 mL/1.6 mL). After RP-flash chromatography, the protected cyclic peptide **30** (9.7 mg, yield 69%) was obtained as a white solid. MS (ESI^+^) *m*/*z* 762.4 [M + H]^+^. Compound **14** was prepared according to the deprotection general procedure. The protected cyclic intermediate **30** (9.7 mg, 0.013 mmol, 1 equiv) was treated with 0.64 mL of TFA/TIS/H_2_O (95:2.5:2.5) mixture and, after RP-flash chromatography, cyclic peptide **14** (5.3 mg, yield 60%) was obtained as a white glassy solid. MS (ESI^+^) *m*/*z* 706.4 [M + H]^+^. ^1^H-NMR (MeOD, 400 MHz): *δ* 7.64 (dd, *J* = 8.0, 1.4 Hz, 1H, H4′ MPUPA), 7.40 (m, 2H, H3′ MPUPA), 7.22 (m, 2H, H2′ MPUPA), 7.18 (m, 1H, H5′ MPUPA), 7.13 (d, *J* = 8.5 Hz, 1H, H7′ MPUPA), 7.04 (ddd, *J* = 7.5, 7.5, 1.0 Hz, 1H, H6′ MPUPA), 4.67 (m, 1H, H4 Amp), 4.63 (d, *J* = 9.4 Hz, 1H, H2 Amp), 4.48 (t, *J* = 7.6 Hz, 1H, Hα Asp), 4.07 (t, *J* = 8.0 Hz, 1H, Hα Leu), 3.91 (dd, *J* = 11.9, 6.1 Hz, 1H, H5 Amp), 3.78 (d, *J* = 6.5 Hz, 1H, Hα Val), 3.70 (s, 2H, H1′ MPUPA), 3.68 (m, 1H, H5 Amp), 3.00 (dd, *J* = 16.6, 7.5 Hz, 1H, Hβ Asp), 2.90 (dd, *J* = 16.6, 7.5 Hz, 1H, Hβ Asp), 2.50 (m, 1H, Hβ Val), 2.46 (m, 1H, H3 Amp), 2.31 (s, 3H, H8′ MPUPA), 2.08 (d, *J* = 14 Hz, 1H, H3 Amp), 1.73 (m, 1H, Hγ Leu), 1.58 (m, 2H, Hβ Leu), 0.96 (m, 12H, 2CH_3_ Val, 2CH_3_ Leu). [α]_D_^25^: −38 (*c* 1.0, MeOH).

#### 4.3.15. *c*[(MPUPA)Amp-Leu-Glu-Val] (**15**)

Compound **31** was prepared according to the cyclization general procedure. A solution of linear peptide **24** (15.4 mg, 0.019 mmol) and 2,4,6-collidine (7.7 µL, 0.058 mmol) in dry DCM/DMF solvent mixture (13.4 mL/0.6 mL) was added dropwise to a solution of HATU (22.1 mg, 0.058 mmol) and HOAt (7.9 mg, 0.058 mmol) in dry DCM/DMF solvent mixture (10.0 mL/1.0 mL). After RP-flash chromatography, the protected cyclic peptide **31** (9.8 mg, yield 65%) was obtained as a white solid. MS (ESI^+^) *m*/*z* 776.4 [M + H]^+^. Compound **15** was prepared according to the deprotection general procedure. The protected cyclic intermediate **31** (9.8 mg, 0.01 mmol) was treated with 0.55 mL of TFA/TIS/H_2_O (95:2.5:2.5) mixture and then, after RP-flash chromatography, cyclic peptide **15** (5.1 mg, yield 56%) was obtained as a white glassy solid. MS (ESI^+^) *m*/*z* 720.4 [M + H]^+^.^1^H-NMR (MeOD, 400 MHz): *δ* 7.64 (d, *J* = 8.0 Hz, 1H, H4′ MPUPA), 7.41 (d, *J* = 8.5 Hz, 2H, H3′ MPUPA), 7.21 (d, *J* = 8.6 Hz, 2H, H2′ MPUPA), 7.18 (dd, *J* = 7.9, 7.9 Hz, 1H, H5′ MPUPA), 7.13 (d, *J* = 8.5 Hz, 1H, H7′ MPUPA), 7.04 (dd, *J* = 7.3, 7.3 Hz, 1H, H6′ MPUPA), 4.71 (m, 1H, H2 Amp), 4.65 (m, 1H, H4 Amp), 4.60 (m, 1H, Hα Glu), 4.03 (m, 2H, Hα Leu, Hα Val), 3.93 (dd, *J* = 11.4, 6.3 Hz, 1H, H5 Amp), 3.70 (s, 2H, H1′ MPUPA), 3.67 (m, 1H, H5 Amp), 2.47 (m, 1H, H3 Amp), 2.47 (m, 1H, Hβ Val), 2.36 (m, 2H, Hγ Glu), 2.31 (s, 3H, H8′ MPUPA), 2.18 (m, 2H, Hβ Glu), 2.07 (d, *J* = 14.2 Hz, 1H, H3 Amp), 1.69 (m, 3H, Hβ Leu, Hγ Leu), 0.96 (m, 12H, CH_3_ Leu, CH_3_ Val). [α]_D_^25^: −19 (*c* 1.0, MeOH).

#### 4.3.16. *c*[(MPUPA)Amp-Val-Asp-Leu] (**16**)

Compound **34** was prepared according to the cyclization general procedure. A solution of linear peptide **27** (16.1 mg, 0.021 mmol) and 2,4,6-collidine (8.2 µL, 0.062 mmol) in dry DCM/DMF solvent mixture (10 mL/1.0 mL) was added dropwise to a solution of HATU (23.5 mg, 0.062 mmol) and HOAt (8.4 mg, 0.062 mmol) in dry DCM/DMF solvent mixture (15 mL/1 mL). After RP-flash chromatography, the protected cyclic peptide **34** (11.4 mg, yield 73%) was obtained as a white solid. MS (ESI^+^) *m*/*z* 762.4 [M + H]^+^. Compound **16** was prepared according to the deprotection general procedure. The protected cyclic intermediate **34** (11.4 mg, 0.015 mmol) was treated with 0.75 mL of TFA/TIS/H_2_O (95:2.5:2.5) mixture and, after RP-flash chromatography (*R_t_* = 24.3 min), cyclic peptide **16** (4.4 mg, yield 23%) was obtained as a white glassy solid. MS (ESI^+^) *m*/*z* 706.4 [M + H]^+^. ^1^H-NMR (400 MHz, MeOD): *δ* 7.64 (dd, *J* = 8.0, 1.4 Hz, 1H, H4′ MPUPA), 7.40 (m, 2H, H3′ MPUPA), 7.22 (m, 2H, H2′ MPUPA), 7.18 (m, 1H, H5′ MPUPA), 7.13 (d, *J* = 8.5 Hz, 1H, H7′ MPUPA), 7.04 (ddd, *J* = 7.5, 7.5, 1.0 Hz, 1H, H6′ MPUPA), 4.69 (d, *J* = 9.6 Hz, 1H, H4 Amp), 4.68 (m, 1H, Hα Asp), 4.62 (dd, *J* = 8.9, 6.5 Hz, 1H, H2 Amp), 3.90 (dd, *J* = 11.9, 6.4 Hz, 1H, Hβ Asp), 3.82 (m, 1H, Hα Leu), 3.74 (dd, *J* = 8.5, 4.0 Hz, 1H, Hα Val), 3.69 (s, 2H, H1′ MPUPA), 3.67 (d, *J* = 11.9 Hz, 1H, Hβ Asp), 3.00 (dd, *J* = 17.0, 8.8 Hz, 1H, H5 Amp), 2.69 (dd, *J* = 17.0, 6.4 Hz, 1H, H5 Amp), 2.48 (ddd, *J* = 14.3, 10.3, 6,7 Hz, H3 Amp), 2.31 (s, 3H, H8′ MPUPA), 2.31 (m, 1H, Hβ Leu), 2.11 (d, *J* = 14.2 Hz, 1H, H3 Amp), 1.98 (m, 1H, Hβ Val), 1.87 (m, 1H, Hβ Leu), 1.70 (m, 1H, Hγ Leu), 1.10 (d, *J* = 6.8 Hz, 3H, CH_3_ Val), 1.02 (d, *J* = 6.8 Hz, 3H, CH_3_ Val), 0.92 (d, *J* = 6.8 Hz, 6H, 2CH_3_ Leu). [α]_D_^25^: −24 (*c* 1.0, MeOH).

#### 4.3.17. *c*[(MPUPA)Amp-Val-Glu-Leu] (**17**)

Compound **35** was prepared according to the cyclization general procedure. A solution of linear peptide **28** (17.0 mg, 0.02 mmol) and 2,4,6-collidine (8.7 µL, 0.064 mmol) in dry DCM/DMF solvent mixture (10.0 mL/2 mL) was added dropwise to a solution of HATU (24.4 mg, 0.064 mmol) and HOAt (8.7 mg, 0.064 mmol) in dry DCM/DMF solvent mixture (16.0 mL/1 mL). After RP-flash chromatography, the protected cyclic peptide **35** (12.0 mg, yield 73%) was obtained as a white solid. MS (ESI^+^) *m*/*z* 776.4 [M + H]^+^. Compound **17** was prepared according to the deprotection general procedure. The protected cyclic intermediate **35** (12.0 mg, 0.016 mmol) was treated with 0.77 mL of TFA/TIS/H_2_O (95:2.5:2.5) mixture and, after RP-flash chromatography, cyclic peptide **17** (13.7 mg, yield 33%) was obtained as a white glassy solid. MS (ESI^+^) *m*/*z* 720.3 [M + H]^+^. ^1^H-NMR (MeOD, 400 MHz): *δ* 7.64 (dd, *J* = 8.0, 1.4 Hz, 1H, H4′ MPUPA), 7.40 (m, 2H, H3′ MPUPA), 7.22 (m, 2H, H2′ MPUPA), 7.18 (m, 1H, H5′ MPUPA), 7.13 (d, *J* = 8.5 Hz, 1H, H7′ MPUPA), 7.04 (ddd, *J* = 7.3, 7.3, 1.1 Hz, 1H, H6′ MPUPA), 4.74 (m, 1H, H4 Amp), 4.68 (d, *J* = 9.8 Hz, 1H, H2 Amp), 4.31 (t, *J* = 8.4 Hz, 1H, Hα Glu), 3.93 (dd, *J* = 11.4, 6.3 Hz, 1H, H5 Amp), 3.85 (m, 1H, Hα Leu), 3.71 (m, 1H, Hα Val), 3.69 (s, 2H, H1′ MPUPA), 3.69 (m, 1H, H5 Amp), 2.48 (m, 1H, H3 Amp), 2.31 (s, 3H, H8′ MPUPA), 2.31 (m, 2H, Hγ Glu), 2.07 (m, 1H, H3 Amp), 2.02 (m, 2H, Hβ Glu), 2.01 (m, 1H, Hβ Val), 1.77 (m, 2H, Hβ Leu), 1.61 (m, 1H, Hγ Leu), 1.10 (m, 6H, 2CH_3_ Val), 0.93 (m, 6H, 2CH_3_ Leu). [α]_D_^25^: −38 (*c* 1.0, MeOH).

#### 4.3.18. *c*[(MPUPA)Amp-Arg-Gly-Asp] (**18**)

Compound **36** was prepared according to the cyclization general procedure. A solution of linear peptide **29** (10.1 mg, 0.001 mmol) and 2,4,6-collidine (3.8 µL, 0.029 mmol) in dry DCM/DMF solvent mixture (11.2 mL/0.8 mL) was added dropwise to a solution of HATU (10.9 mg, 0.04 mmol) and HOAt (3.9 mg, 0.029 mmol) in dry DCM/DMF solvent mixture (6.2 mL/0.4 mL). After RP-flash chromatography, the protected cyclic peptide **36** (5.0 mg, yield 43%) was obtained as a white solid. MS (ESI^+^) *m*/*z* 1028.5 [M + H]^+^. Compound **18** was prepared according to the deprotection general procedure. The protected cyclic intermediate **36** (5.0 mg, 0.01 mmol) was treated with 0.22 mL of TFA/TIS/H_2_O (95:2.5:2.5) mixture and, after RP-flash chromatography, cyclic peptide **18** (3.5 mg, yield 63%) was obtained as a yellowish glassy solid. MS (ESI^+^) *m*/*z* 707.3 [M + H]^+^.^1^H-NMR (MeOD, 400 MHz): *δ* 7.64 (d, *J* = 8.0 Hz, 1H, H4′ MPUPA), 7.41 (d, *J* = 8.5 Hz, 2H, H3′ MPUPA), 7.21 (d, *J* = 8.6 Hz, 2H, H2′ MPUPA), 7.18 (dd, *J* = 7.9, 7.9 Hz, 1H, H5′ MPUPA), 7.13 (d, *J* = 8.5 Hz, 1H, H7′ MPUPA), 7.04 (dd, *J* = 7.3, 7.3 Hz, 1H, H6′ MPUPA), 4.71 (t, *J* = 5.6 Hz, 1H, Hα Asp), 4.66 (d, *J* = 9.5 Hz, 1H, H2 Amp), 4.59 (t, *J* = 6.0 Hz, 1H, H4 Amp), 4.19 (d, *J* = 13.8 Hz, 1H, Hα Gly), 4.09 (t, *J* = 6.8 Hz, 1H, Hα Arg), 3.93 (dd, *J* = 11.8, 6.4 Hz, 1H, H5 Amp), 3.70 (m, 3H, H1′ MPUPA, H5 Amp), 3.42 (d, *J* = 13.8 Hz, 1H, Hα Gly), 3.20 (m, 2H, Hδ Arg), 2.81 (d, *J* = 5.7 Hz, 2H, Hβ Asp), 2.50 (m, 1H, H3 Amp), 2.31 (s, 3H, H8′ MPUPA), 2.18 (d, *J* = 14.4, 1H, H3 Amp), 1.76 (m, 2H, Hβ Arg), 1.65 (m, 2H, Hγ Arg). [α]_D_^25^: −38 (*c* 1.0, MeOH).

### 4.4. Biology

#### 4.4.1. Cell Culture

Jurkat E6.1 cells were purchased from American Type Culture Collection (ATCC, Rockville, MD, USA) and were routinely cultured in RPMI-1640 (LifeTechnologies, Milan, Italy) supplemented with 10% FBS (fetal bovine serum, Life Technologies) and 2 mM glutamine. Cells were kept at 37 °C under 5% CO_2_ humidified atmosphere. Jurkat cells are a widely used cell model to study potential agonist or antagonist ligands able to modulate integrin-mediated cell adhesion [46,48,49,50,51]. Jurkat cells endogenously express α_4_β_1_ integrin [13].

#### 4.4.2. Cell Adhesion Assays

The assays were performed as previously described [51]. In brief, black 96 well plates were coated with VCAM-1 (2 μg/mL) overnight at 4 °C; then, non-specific hydrophobic binding sites were blocked with 1% BSA (bovine serum albumin, Sigma-Aldrich) in HBSS (Life Technologies) for 30 min at 37 °C. Jurkat cells were labelled with CellTracker green CMFDA (12.5 μM, Life Technolgies) and pre-incubated with various concentration (10^−4^–10^−10^ M) of each new cyclopeptidomimetic or with the vehicle (DMSO) for 30 min at 37 °C. Afterwards, Jurkat cells were plated on VCAM-1-coated wells and incubated for 30 min at 37 °C. Then, wells were washed three times with 1% BSA in HBSS and Jurkat cells were lysed with 0.5% Triton X-100 in PBS for 30 min at 4 °C. Green fluorescence (Ex485 nm/Em 535 nm) was measured in an EnSpire Multimode Plate Reader (PerkinElmer, Waltham, MA, USA). Experiments were performed in quadruplicate and repeated at least three times. The number of adherent cells was determined by comparison with a standard curve made with a known concentration of labelled Jurkat cells. Data analysis and IC_50_/EC_50_ values were calculated using GraphPad Prism 6.0 (GraphPad Software, San Diego, CA, USA).

In another set of experiments, Jurkat cells were plated (500,000 cells/well) in 96-wells plate previously coated by passive absorption with VCAM-1 (2 μg/mL) or with compound **16** (10 μg/mL), the most effective compound under examination, or with BSA (10 μg/mL, as negative control). To investigate any potential ligand binding competition, 30 min before plating cells on VCAM-1-coated wells, BIO1211 (1 μM) was added to the cells pre-incubated with compound **16** (100 μM) or compound **16** was used to treat to the cells pre-incubated with BIO1211. Moreover, Jurkat cells pre-incubated with different concentrations of BIO1211 (100 mM–10 nM) were plated in compound **16**-coated wells. The number of adherent cells was determined as described above.

## 5. Conclusions

The central role played by α_4_β_1_ integrin in inflammatory and autoimmune pathologies and tumor-related diseases has been widely explored, so the search for potent and selective α_4_β_1_ integrin binders has been and remains a topic of interest in current biomedical research.

The present study, addressing the synthesis of new potential α_4_β_1_ integrin ligands, is rooted in this challenging field of research. Based on some initial computational suggestions, seven new cyclic peptidomimetics, all bearing a common aminoproline core scaffold and an MPUPA hexocyclic motif, were synthesized and structurally characterized. Preliminary in vitro biological evaluation revealed that one of these candidates, compound **16**, featuring the constrained *c*(Amp-VDL) cyclotetrapeptide structure, showed a moderate ability to enhance Jurkat cell adhesion to VCAM-1, and further biological evidence pointed to the exclusion of competition with the known antagonist BIO1211 for the same receptor binding site.

Further biological investigations will be necessary for a complete characterization of the agonist behavior of our compounds, to assess integrin selectivity and possibly define structural requirements for agonist vs. antagonist activity, with the ultimate intention of contributing to the expanding knowledge in the field of small-molecule integrin ligands.

## Figures and Tables

**Table 1:** Effects of the new Amp-based cyclopeptides on α_4_β_1_ integrin-mediated cell adhesion.

Compound	Structure ^[a]^	EC_50_/IC_50_ (μM) ^[b]^
**12**	*c*[Amp(MPUPA)Leu-Asp-Val-Gly]	>100
**13**	*c*[Amp(MPUPA)Leu-Glu-Val-Gly]	>100
**14**	*c*[Amp(MPUPA)Leu-Asp-Val]	>100
**15**	*c*[Amp(MPUPA)Leu-Glu-Val]	>100
**16**	*c*[Amp(MPUPA)Val-Asp-Leu]	0.37 ± 0.09 *agonist*
**17**	*c*[Amp(MPUPA)Val-Glu-Leu]	>100
**18**	*c*[Amp(MPUPA)Arg-Gly-Asp]	>100
**1**	MPUPA-Leu-Asp-Val-Pro	0.0046 ± 0.0030 ^[c]^ *antagonist*

^[a]^ MPUPA = 4-[(*N*-2-Methylphenyl)ureido]phenylacetyl. ^[b]^ Data are presented as EC_50_ for compounds enhancing cell adhesion (*agonists*) and as IC_50_ for compounds reducing cell adhesion (*antagonists*) (μM). Cell adhesion mediated by α_4_β_1_ integrin was measured by assaying Jurkat cell adhesion to VCAM-1 (2 μg/mL). Values are the means ± SD, n = 3. ^[c]^ Value determined in this assay. For a previously reported value, see ref. [13].

## Data Availability

Authors will release data of docking complex results upon request. Please, contact the corresponding author.

## Sample Availability

Samples of the final compounds **12**–**18** are available from the authors.
